# The social-sensory interface: category interactions in person perception

**DOI:** 10.3389/fnint.2012.00081

**Published:** 2012-10-17

**Authors:** Jonathan B. Freeman, Kerri L. Johnson, Reginald B. Adams, Nalini Ambady

**Affiliations:** ^1^Department of Psychological and Brain Sciences, Dartmouth CollegeHanover, NH, USA; ^2^Department of Communication Studies, University of California, Los AngelesLos Angeles, CA, USA; ^3^Department of Psychology, University of California, Los AngelesLos Angeles, CA, USA; ^4^Department of Psychology, The Pennsylvania State UniversityUniversity Park, PA, USA; ^5^Department of Psychology, Stanford UniversityStanford, CA, USA

**Keywords:** person perception, visual perception, face perception, social categorization

## Abstract

Research is increasingly challenging the claim that distinct sources of social information—such as sex, race, and emotion—are processed in discrete fashion. Instead, there appear to be functionally relevant interactions that occur. In the present article, we describe research examining how cues conveyed by the human face, voice, and body interact to form the unified representations that guide our perceptions of and responses to other people. We explain how these information sources are often thrown into interaction through bottom-up forces (e.g., phenotypic cues) as well as top-down forces (e.g., stereotypes and prior knowledge). Such interactions point to a person perception process that is driven by an intimate interface between bottom-up perceptual and top-down social processes. Incorporating data from neuroimaging, event-related potentials (ERP), computational modeling, computer mouse-tracking, and other behavioral measures, we discuss the structure of this interface, and we consider its implications and adaptive purposes. We argue that an increased understanding of person perception will likely require a synthesis of insights and techniques, from social psychology to the cognitive, neural, and vision sciences.

With only a fleeting glimpse, a constellation of near-instant judgments are often made about another person. Although frequently warned not to “judge a book by its cover,” our tendency to make meaning out of the sensory information availed by others is typically beyond our conscious control. From minimal cues afforded by the face, voice, and body, we unwittingly infer the intentions, thoughts, personalities, emotions, and category memberships (e.g., sex, race, and age) of those around us. While some of these judgments may be expectancy-driven and biased by our stereotypes (Brewer, 1988; Fiske and Neuberg, 1990; Macrae and Bodenhausen, 2000), others may be surprisingly accurate and expose humans' exquisite ability to perceive other people from only the briefest of observations (Ambady et al., 2000; Willis and Todorov, 2006).

This astounding ability to perceive other people, however, is plagued by a basic contradiction. As readily and rapidly as we may dispel our judgments of others, each judgment requires an astonishing complexity of mental processing. Despite their complexity, however, they occur with remarkable ease. From a single face, for example, any numbers of perceptions (e.g., sex and emotion) are immediately available, but each requires the integration of an enormous amount of information. Unlike objects, other people are highly complex stimuli, embedded in a rich set of contexts and grounded in multiple sensory modalities. All the features and configural properties of a person's face must be bound together, along with that person's hair and array of bodily cues. Auditory cues of a person's voice are available as well, and these must be bound together with the person's visual cues to form a coherent social percept. Such a complexity of bottom-up sensory information is matched, however, by a similar complexity in top-down information sources that are uniquely present in person perception. For example, people bring a great deal of prior knowledge, stereotypic expectations, and affective and motivational states to the process of perceiving others. The influences of these top-down factors may often seep down into the perceptual process itself. How does such a vast array of information—both bottom-up and top-down—rapidly conspire to drive perception in the very short time it takes to arrive at an instant judgment of another person?

In this article, we first provide background on person perception from the perspective of social psychology followed by background from the perspective of the cognitive, vision, and neural sciences. We then describe how these literatures have traditionally converged on the argument for non-interactive processing of different category dimensions. We then discuss more recent evidence for category interactions, either through top-down (e.g., driven by stereotypic expectations) or bottom-up (e.g., driven by perceptual cues) mechanisms. Finally, we explain how a recent framework and model of person perception can capture such effects and potentially yield a clearer picture of person perception. When then finish with some concluding remarks.

## Social psychology on person perception

If person perception is characterized, on the one hand, by being highly complex, and on the other by being highly efficient, social psychological research has historically placed a great deal of focus on the latter. Seminal work in social psychology by Allport (1954), Sherif (1967), and Tajfel (1969), for example, argued that individuals perceive others via spontaneous, perhaps inevitable, category-based impressions that are highly efficient and designed to economize on mental resources. Since then, a vast array of studies has demonstrated that such category-based impressions bring about a host of cognitive, affective, and behavioral outcomes. Mere activation of a social category, it has been shown, readily changes how individuals think about others, feel about them, and behave toward them, often in ways that may operate non-consciously (e.g., Brewer, 1988; Devine, 1989; Fiske and Neuberg, 1990; Gilbert and Hixon, 1991; Bargh, 1994, 1999; Fazio et al., 1995; Dovidio et al., 1997; Sinclair and Kunda, 1999). A strong emphasis in social psychology, therefore, has been to document the downstream implications of person categorization and its myriad outcomes for social interaction.

With a focus on subsequent interpersonal phenomena, a clear research strategy emerged in the literature. Often, a single category of interest would be isolated (e.g., race) and its influences on subsequent behavior measured, while all other categories were controlled (e.g., sex, age, and emotion). This afforded tremendous insights into the downstream dynamics of single categorizations, but it lacked breadth in understanding the complexity of real-world categorization. In reality, social targets may be categorized along any number of possible dimensions. Of the many potential categories, then, which get the privilege of perceivers' processing, and which are thrown aside? A prevalent answer to this question has been that one category (e.g., race) comes to dominate perception, while all others (e.g., sex and age) are inhibited from working memory (Macrae et al., 1995; Sinclair and Kunda, 1999). Presumably, this category selection process makes the perceiver's job easier (e.g., Bodenhausen and Macrae, 1998), keeping with the longstanding notion that social categorization is for maximizing cognitive efficiency (Allport, 1954). Although this perspective has been valuable, its upshot has been a tendency to view each category membership as isolated and independent (with one dominating and all others cast aside), and to neglect targets' multiple simultaneous memberships and how they may, in some cases, interact.

Such multiple social categorization is one of the most fascinating and distinctive aspects of person perception. For instance, whereas the perception of an object generally affords only one focal type of construal (e.g., “that's a table”), multiple construals are simultaneously available when perceiving other people and each is highly relevant. A single face stimulus, for example, permits a rich array of judgments, including basic categories (e.g., sex, race, age, and emotion), perceptually ambiguous categories (e.g., sexual orientation), personality traits (e.g., warmth and competence), intentions (e.g., deception), among many others. Although the results of studies examining personality judgments have long implied that they may occur in parallel (e.g., Ambady et al., 2000; Todorov and Uleman, 2003; Willis and Todorov, 2006), the underlying basis of the parallelism and the mutual influences each judgment may exert on one another have rarely been investigated. With respect to basic social categories, parallel memberships has been examined in the context of high-level impressions, social reasoning, and memory (e.g., Strangor et al., 1992; Kunda and Thagard, 1996; Vescio et al., 2004; Smith, 2006; Crisp and Hewstone, 2007), but their simultaneous interplay has been scarcely considered in lower-level sensory-based perceptions. Thus, although construing others is uniquely characterized by an enormous number of simultaneously available perceptions, the social literature has tended to overlook their compound nature and how they might interact.

## Cognitive, vision, and neural sciences on person perception

In the cognitive face-processing literature, non-interactive processing has even been argued formally. Such work has been largely guided by the cognitive architecture laid out in the influential Bruce and Young (1986) model of face perception, which proposed a dual processing route. Initially, a structural encoding mechanism constructs a representation of a face's features and configuration. Processing results from structural encoding are then sent down two non-interactive, functionally independent routes. One route works on processing a target's static cues, such as identity, while a separate route works on processing more complex and dynamic cues, including emotion expressions, speech, and other “visually derived semantic information,” such as social categories. Haxby and colleagues (2000) extended the Bruce and Young model to the neural level. They proposed that, first the early perception of facial features is mediated by the inferior occipital gyrus (IOG) (analogous to Bruce and Young's structural encoding mechanism). The labor is then divided onto the lateral fusiform gyrus, which processes static cues such as identity, and the superior temporal sulcus (STS), which processes dynamic cues such as emotion expressions (Haxby et al., 2000). The model was supported by a number of fMRI studies demonstrating that fusiform regions tend to be more sensitive to identity, whereas the STS tends to be more sensitive to emotion (LaBar et al., 2003; Winston et al., 2004). Additional evidence came from lesion studies, which showed that distinct lesions correspond with selective impairments in processing identity versus emotion expressions (Tranel and Damasio, 1988; Young et al., 1993). A popular view, therefore, has been that the processing of multiple dimensions, such as identity and emotion, run independently and in parallel. As such, although multiple dimensions may be processed simultaneously, their processing is not generally thought to cross paths.

In contrast, a growing body of research emerging from the vision sciences has found a great deal of evidence for interdependence in processing various facial dimensions. Using selective attention paradigms such as the Garner interference paradigm (Garner, 1976), a number of studies have tested perceivers' ability to selectively attend to one dimension (e.g., facial identity) while ignoring task-irrelevant dimensions (e.g., facial emotion). Over the years, researchers have reported interference effects for many facial dimensions, including sex and emotion (Atkinson et al., 2005), sex and age (Quinn and Macrae, 2005), identity and sex (Ganel and Goshen-Gottstein, 2004), identity and emotion (Schweinberger et al., 1999), eye gaze and emotion (Graham and LaBar, 2007), and sex and race (Johnson et al., in press). Such findings suggest interdependence among various facial dimensions, casting doubt on the traditional view that the processing of various facial dimensions is strictly separated. Calder and Young (2005) proposed a principal component analysis (PCA) framework for face perception, accounting for such interference effects by way of a single multidimensional face-coding system. According to their framework, neural dissociations typically taken as evidence for distinct processing pathways (e.g., identity processed via fusiform regions and emotion processed via the STS) reflect statistical regularities inherent in the visual input itself, rather than separate neural structures dedicated for particular facial dimensions. Such work presents serious challenges for the traditional view that the processing of one facial dimension is insulated from the processing of all other dimensions.

Additional evidence for inherent inseparability between multiple facial dimensions comes from neuronal recordings in non-human primates. In monkey temporal cortex, for example, there are groups of neurons that are sensitive to the conjunction of both identity and emotion, as well as the conjunction of eye gaze and emotion (Hasselmo et al., 1989; Perrett et al., 1992). There also appears to be a temporal evolution in how these neurons represent aspects of facial information. In one study, the transient response of face-sensitive temporal cortex neurons was found to initially reflect a rough, global discrimination of a visual stimulus as a face (rather than some other shape). Subsequent firing of this same neuronal population, however, appeared to sharpen over time by coming to represent finer facial information, such as emotion expression and, slightly later, facial identity (Sugase et al., 1999). In humans, studies recording event-related potentials (ERP) also suggest a dynamic evolution of face representation, from more global (structural) encoding of the face to finer-grained information, such as sex category (Freeman et al., 2010). Such findings suggest that there are overlapping neuronal populations involved in encoding multiple aspects of facial information. Taken together with the interference effects above, it appears that the processing of a single facial dimension may, at least in some cases, be neurally coextensive with the processing of other dimensions, and may readily interact and influence those other dimensions.

## Combinatorial person perception

Evidence that the perceptual processing of a social target's various identities may be coextensive during perception implies that those identities may be thrown into interaction. Thus, the dynamics of social perceptions raise the intriguing possibility that the perception of multiple social categories and transient states are not only coactive during perception, but that they also are mutually dependent upon one another. As such, social perceptions are combinatorial. The perception of one social category may systematically facilitate or inhibit the perception of another social category. Such impacts appear to occur via two distinct routes—one through the top-down influence of factors that originate in the perceiver (e.g., existing knowledge structures and motivations) and one through the bottom-up influence of factors that originate in the target of perception (e.g., overlapping visual cues). Next we review evidence supporting these two routes by which complexities in both the perceiver and the percept are likely to impact perceptions and their efficiency. Then, we review work that examines the underlying cognitive and neural processing through which these two forms of influence dynamically collaborate to yield a coherent and adaptively sensitive social percept.

### Top-down perceiver impacts

Some factors that impinge on the combinatorial nature of social perception originate in the perceiver. Although perception in general was long presumed to be impenetrable to and isolated from higher-order cognitive processes, recent evidence suggests otherwise. Instead, low-level sensory processes may be modulated by social cognitive factors (e.g., Bar, 2004; Amodio and Frith, 2006; Kveraga et al., 2007), and this is apparent at the behavioral and neural levels. For instance, interconnectivity has been identified between the amygdala and the STS, tethering brain regions responsible for processing emotion content and the visual analysis of human actions, respectively (Amaral et al., 2003). In terms of functionally adaptive face processing, the amygdala, orbitofrontal cortex (OFC), and STS form a three-node pathway that has been referred to as the “social brain” (Brothers, 1990) important for processing social and emotional meaning from the face. Pathways from the STS to the amygdala support adaptive behavioral response to biological movement including facial expression and looking behavior (Aggleton et al., 1980; Brothers and Ring, 1993) and pathways to the OFC support adaptive behavioral responding, conceptual knowledge retrieval, and decision making during the processing of emotion information (Bechara et al., 2000). Beyond these connections, the amygdala is also densely interconnected with regions involved in affective, cognitive, perceptual, and behavioral responses to faces where exteroceptive and interoceptive information can be integrated, and it is known to exert top-down modulation on extrastriate responses (Adolphs, 2003). Specifically, the amygdala is known to be reciprocally connected to regions involved in face perception, such as the IOG, which is involved in low-level structural encoding of faces (Haxby et al., 2000), and the fusiform gyrus, which is thought to be specialized for identity processing (Kanwisher, 2000; Kanwisher and Yovel, 2006). As such, it appears to act as an integrative center for the processing and relaying of socially relevant facial information.

The bidirectional and dynamic nature of the neural processing subserving social perception opens up the opportunity for social perceptions to be modulated by factors that are inherent to the perceiver, including existing knowledge structures (i.e., stereotypes) and current motivation states. Indeed, mounting evidence demonstrates that such factors impact social perception systematically, leading to functional biases or attunements in perceptions of the world and the people within it.

A perceiver's knowledge structures may impact perception through expectations. The social categories to which people belong each activate a network of knowledge structures that are associated with the particular category (Bargh, 1999; Devine, 1989). For instance, perceiving the category male is likely to elicit stereotypes of assertiveness and strength (Hess et al., 2010); likewise perceiving an emotion such as anger may facilitate activation of the sex category male (Hess et al., 2009). Once these knowledge structures are activated, they are thought to have a pronounced impact on basic perceptual processes (Freeman and Ambady, 2011a).

Indeed, recent evidence suggests that stereotyped expectations that are elicited from cues to a social category can bias low-level aspects of perception. Race-cuing features, for instance, alter judgments of a target's skin tone (Levin and Banaji, 2006). When facial cues implied a Black identity, participants were prone to overestimate the pigmentation of a target's face; when facial cues implied a White identity, in contrast, participants underestimated the pigmentation of a target's face. Thus, social category knowledge structures biased the luminance properties of a face. In other research, race-cuing hairstyles led perceivers to disambiguate the race of an otherwise race-ambiguous face in a category-consistent manner (MacLin and Malpass, 2001, 2003). Not only did these race categories influence memory for the faces, but as was found in the study above, race-cuing hairstyles also influenced low-level aspects of perception. Black faces were perceived to have a darker skin tone, wider faces and mouths, and less protruding eyes, relative to Hispanic faces.

Similarly, stereotyped expectations elicited from cues to a social category can bias perceptions. For instance, Johnson et al. (in press) demonstrated that sex categorizations and their efficiency were influenced by race-category membership. Male categorizations were more efficient for Black faces, but less efficient for Asian faces; female categorizations, in contrast, were more efficient for Asian faces, but less efficient for Black faces. These results were obtained, in part, because the stereotypes associated with race and sex are substantively overlapping. For example, both Black individuals and men are stereotypically associated as aggressive; both Asian individuals and women are stereotypically associated as docile. In another series of studies, contextual cues surrounding a face were found to alter race perception via stereotypes. If a face was surrounded by business attire, it was more likely to be perceived as White (as businesspeople and White people are both stereotypically associated as high status); when surrounded by janitor attire, the face was more likely to be perceived as Black (as janitors and Black people are both stereotypically associated as low status). These effects of stereotypes became more pronounced as the face's race increased in ambiguity. Further, even when a participant's ultimate response was not biased by the context and by stereotypes, their hand movement en route to the response often swerved nevertheless to the opposite response stereotypically associated with the attire (Freeman et al., 2011). Thus, even in cases where stereotypes do not exert an influence on a perceptual outcome, they may still substantially alter the perceptual process.

Additionally, a perceiver's motivation state may alter perceptual processing. Visual cues to identity are potent sources of information that, under many circumstances, compel surprisingly accurate social judgments (Ambady and Rosenthal, 1992). At times, however, observers' judgments are prone to functional perceptual biases (Haselton and Nettle, 2006). From this perspective, perceptual judgments are always rendered with some degree of uncertainty, and the relative costs associated with various errors are likely to be asymmetric. Motivational factors, therefore, will tend to bias the perceptions of the physical world in a manner that minimizes potential costs to the perceiver. Race-category labels that are paired with otherwise race-ambiguous faces change how a face is processed (Corneille et al., 2004; Michel et al., 2007) and determine whether a face will be remembered (see also Pauker and Ambady, 2009; Pauker et al., 2009), in ways that appear to be, at least in part, motivationally driven (see also Sacco and Hugenberg, 2009). Perceivers are also likely to categorize targets to be Black—a social category that is stereotyped to be dangerous—when personal safety is a concern (Miller et al., 2010), and perceivers who are high in racial prejudice are also more likely to categorize ambiguous race faces as Black (Hugenberg and Bodenhausen, 2004). A motivation to identify coalitional alliances has been identified as a functional underpinning for race categorization (Kurzban et al., 2001).

Sex categorizations show a similar pattern of functionally biased perceptions. Because men overall tend to be physically larger and stronger than women, they pose a greater potential threat to perceivers. In any condition of uncertainty, therefore, a functional bias is likely to favor a male percept. In fact, a male categorization has long been argued to comprise the “default” social category judgment (Zarate and Smith, 1990; Stroessner, 1996). Under conditions that may signal potential threat, this tendency appears to be exacerbated. When categorizing the sex of bodies, for example, perceivers show a pronounced male categorization bias for every body shape that is, in reality, not exclusive to women (Johnson et al., in press), and this tendency is most pronounced when perceivers are in a fearful state. Similarly, point-light defined arm motions that depict a person throwing an object are overwhelmingly categorized as male when the person engages a threatening emotion state (i.e., anger), relative to any other emotion state (Johnson et al., 2011). Moreover, the findings from Johnson et al. (in press) are consistent with the notion that Black targets—who are stereotyped as dangerous—are likely to more readily compel male categorizations.

Although perceivers are generally adept in achieving accurate social perception, accuracy goals may sometimes be overshadowed by other motivational concerns (e.g., situational desires or physical safety concerns). In such circumstances, current motivations may outweigh accuracy objectives, leading social perceptions to be functionally biased in a directional fashion. In sum, both perceptual attunements and functional biases may emerge from the top-down modulation of social perception, either through motivation, existing knowledge structures, or both.^1^

### Bottom-up target impacts

Other factors that impinge on the combinatorial nature of social perception originate in the target of perception. This is because some perceptual attunements and biases are driven by the incoming sensory information itself. As such, cues to social identities may be confounded at the level of the stimulus. Such effects are now well documented for important intersections of social categories including sex and emotion, sex and race, and race and emotion. Importantly, because these categories share cues, their perception becomes inextricably tethered, in turn producing attunements and biases that are moderated by the unique combination of cues and categories.

One particularly intriguing juxtaposition of these dual routes of influence is in the perception of sex and emotion categories. This particular effect has received some attention over the years, initially with respect to shared stereotypes between emotions and sex categories. For instance, for many years, researchers found that facial expressions of emotion were perceived to vary between men and women (Grossman and Wood, 1993; Plant et al., 2000). Ambiguities in emotion expression tended to be resolved in a manner that was consistent with gender stereotypes (Hess et al., 2000), and many interpreted such findings as evidence for a top-down modulation of emotion perception in a manner described above. Thus, the prevailing belief was that common associations between sex and emotion categories lead to biases in perceptual judgments.

More recent research clarified that such results may also emerge via an alternate route. One argument put forth by Marsh et al. (2005) proposed that some facial expressions in humans, in this case anger and fear, evolved to mimic more stable appearance cues related to facial maturity. Likewise, gender appearance is similarly associated with facial features that perceptually overlap with facial maturity (Zebrowitz, 1997). Like facial maturity and masculinized facial features, anger is characterized by a low, bulging brow and small eyes. Conversely, like babyfacedness and feminized features, fear is distinguished by raised and arched brow ridge and widened eyes. Perhaps not too surprisingly then, several studies have hinted at a confounded nature between emotional expression and gender (Hess et al., 2004). One more recent study examined the confound between gender and emotional expression of anger and happiness (Becker et al., 2007). In an even more recent study (Hess et al., 2009), both happy and fearful expressions were found to bias perception of otherwise androgynous faces toward female categorization, whereas anger expressions biased perception toward male categorization.

Such physical resemblance has been revealed in an even more compelling manner through computer-based models that are trained with facial metric data to detect appearance-based and expression cues in faces (e.g., Said et al., 2009; Zebrowitz et al., 2010). Critically, such studies avoid confounds with socially learned stereotypes. In one study, Zebrowitz et al. (2007) trained a connectionist model to detect babyfacedness versus maturity in the face, and then applied this model to detecting such cues in surprise, anger, happy, and neutral expressions. They found that the model was detected babyfacedness in surprise expressions and maturity in anger expressions due to similarities in height of brow. Additionally, the authors found that objective babyfacedness (as determined by the connectionist model) mediated impressions of surprise and anger in those faces reported by human judges. In this way, they were able to provide direct evidence for babyfacedness overgeneralization effects on a wide array of perceived personality traits.

Overlapping perceptual cues affect a number of other category dimensions as well. Some sex and race categories, for example, appear to share overlapping features. In one study using a statistical face model (derived from laser scans of many faces), cues associated with the Black category and cues associated with the male category were found to share a degree of overlap. This, in turn, facilitated the sex categorization of Black men relative to White or Asian men (Johnson et al., in press). A similar overlap exists between eye gaze and emotional expressions. Gaze has the interesting property of being able to offer functional information to a perceiver that, when paired with certain expressions, can lead to interesting interactive effects. According to the shared signal hypothesis (Adams et al., 2003, Adams and Kleck, 2005), cues relevant to threat that share a congruent underlying signal value should facilitate the processing efficiency of an emotion. Because direct and averted eye gaze convey a heightened probability of a target to either approach or avoid a target individual respectively (see Adams and Nelson, 2012, for review), and anger and fear share underlying behavioral intentions (see Harmon-Jones, 2003, for review), this hypothesis suggests that processing should be facilitated when emotion and eye gaze are combined in a congruent manner (i.e., both signaling approach, such as direct-gaze anger and averted-gaze fear) relative to an incongruent manner (i.e., direct-gaze fear and averted-gaze anger).

In support of both functional affordances described above, using speeded reaction time tasks and self-reported perception of emotional intensity, Adams et al. (2003) and Adams and Kleck (2005) found that direct gaze facilitated processing efficiency, accuracy, and increased the perceived intensity of facially communicated approach-oriented emotions (e.g., anger and joy), whereas averted gaze facilitated processing efficiency, accuracy, and perceived intensity of facially communicated avoidance-oriented emotions (e.g., fear and sadness). Similar effects were replicated by Sander et al. (2007) using dynamic threat displays, and by Hess et al. (2007) who found that direct relative to averted anger expressions and averted relative to direct fear expressions elicited more negative responsivity in observers. The converse effect holds as well; facial emotion influences how eye gaze is perceived. Direct eye gaze is recognized faster when paired with angry faces and averted eye gaze is recognized faster when paired with fearful faces (Adams and Franklin, 2009). In addition, perceivers tend to judge eye gaze more often as looking at them when presented on happy and angry faces than neutral or fearful (Lobmaier et al., 2008; Slepian et al., 2011; see also, Martin and Rovira, 1982). Further, (Mathews et al., 2003) found a faster cueing effect (where the attention of an observer is automatically shifted in that a target face is looking) for fear faces than neutral faces for those with high anxiety but not low anxiety, arguably because anxiety increases the observer's attunement to the threat afforded by an expressive display. When eye gaze was shifted dynamically *after* emotion was presented, however, fearful faces were found to induce higher levels of cueing compared to other emotions for all participants regardless of anxiety level (Tipples, 2006; Putman et al., 2007). More recently, Fox et al. (2007) found that fear expressions coupled with averted gaze yielded greater reflexive orienting than did neutral or anger expressions, whereas anger expressions coupled with direct gaze yielded greater attention capture than did neutral or fear expressions. These effects were also moderated by trait anxiety.

On the neural level, gaze has been found to influence amygdala responses to threatening emotion expressions. In an initial study, Adams et al. (2003) found more amygdala response to threat-related ambiguity (i.e., for averted-gaze fear and direct-gaze anger). This study, however, was based on relatively sustained presentations of threat stimuli (2000 ms), whereas some more recent studies have found similar evidence for greater amygdala responses to congruent threat-gaze pairs (direct anger and averted fear) when employing more rapid presentations (Sato et al., 2004; Hadjikhani et al., 2008). Although these latter findings do corroborate Adams et al.'s early behavioral findings for gaze–emotion interactivity, they opened up new questions regarding the role of the amygdala in processing these compound threat cues. In subsequent work, Adams et al. (2012) found direct evidence supporting the conclusion that early, reflexive responses to threat-gaze pairs are more attuned to congruent pairings, whereas later, reflective responses are more attuned to threat-related ambiguity. These differential responses support both an early process that detects threat and sets in motion adaptive responding, but a slightly slower process that is geared to confirming and perpetuating a survival response, or disconfirming and inhibiting an inappropriate response. It is in this interplay of reflexive and reflective processes that threat perception can benefit from different attunements to a threatening stimulus with different but complementary processing demands, to achieve the most timely and adaptive response to other people. In short, many characteristics may interact in person perception because they are directly overlapping, often in functionally adaptive ways.

## The social-sensory interface

The two routes by which social perceptions may be attuned or biased are now well documented, and such research provides an important foundation for understanding the basic mechanisms of social perception. More interesting to our minds is their ability to help us understand how these dual routes work in concert to enable judgments of people that vary along multiple dimensions and across multiple sensory modalities.

Recently, Freeman and Ambady (2011a) proposed a dynamic interactive framework to account for findings such as those reviewed above, and to map out how multiple category dimensions are perceived—and in many cases may interact—in a neurally plausible person perception system. In this system, multiple category dimensions (e.g., sex, race, and emotion) dynamically accumulate evidence in parallel, sometimes in conflicting ways. Importantly, as we will describe shortly, while the system is attempting to stabilize onto particular perceptions over time, it will often throw different category dimensions into interaction with one another. This may occur through either bottom-up or top-down forces, mapping onto the two routes described above. Before describing why and how these interactions would occur, we first outline the structure and function of the system.

Freeman and Ambady (2011a) captured their theoretical system with a computational neural network model. The perceptual process that emerges in this system is a highly integrative one. It incorporates whatever bottom-up evidence is available (from others' facial, vocal, or bodily cues), while also taking into account any relevant top-down sources that could be brought to bear on perception. Thus, the system arrives at stable person construals not only through integrating bottom-up facial, vocal, bodily cues, but also by coordinating with and being constrained by higher-order social cognition (e.g., prior knowledge, stereotypes, motivation, and prejudice). As such, this system permits social top-down factors to fluidly interact with bottom-up sensory information to shape how we see and hear other people. Accordingly, our basic construals of others are always compromises between the sensory information “actually” there and the variety of baggage we bring to the perceptual process. Although traditionally it was long assumed that perception is solely bottom-up and insulated from any top-down influence of higher-order processes (e.g., Marr, 1982; Fodor, 1983), it has become clear that perception arises instead from both bottom-up and top-down influences (e.g., Engel et al., 2001; Gilbert and Sigman, 2007). Thus, we should expect top-down factors to be able to flexibly weigh in on the basic perceptual processing of other people.

In this framework, person perception is treated as an ongoing, dynamic process where bottom-up cues and top-down factors interact over time to stabilize onto particular perceptions (e.g., male or female; Black, White, or Asian). This is because person perception, as implemented in a human brain, would involve continuous changes in a pattern of neuronal activity (Usher and McClelland, 2001; Smith and Ratcliff, 2004; Spivey and Dale, 2006). Consider, for example, the perception of another's face. Early in processing, representations of the face would tend to be partially consistent with multiple categories (e.g., both male and female) because the initial rough “gist” of the face partially supports both categories. As more information accumulates, the pattern of neuronal activity would gradually sharpen into an increasingly confident representation (e.g., male), while other competing, partially-active representations (e.g., female) would be pushed out (Usher and McClelland, 2001; Spivey and Dale, 2006; Freeman et al., 2008). During the hundreds of milliseconds it takes for the neuronal activity to achieve a stable pattern (~100% male or ~100% female), both bottom-up processing of the face as well as top-down factors (e.g., stereotypes) could gradually exert their influences, jointly determining the pattern to which the system gravitates (Grossberg, 1980; Spivey, 2007; Freeman and Ambady, 2011a). Thus, this approach proposes that person perception involves ongoing competition between partially-active categories (e.g., male and female). Further, the competition is gradually weighed in on by both bottom-up sensory cues as well as top-down social factors, until a stable categorization is achieved. Accordingly, bottom-up cues and top-down factors mutually constrain one another to shape person perception.

How might this dynamic social–sensory interface be instantiated at the neural level specifically? Let us consider sex categorization. One possibility is that visual processing of another's face and body in the occipotemporal cortex (e.g., the lateral fusiform gyrus, fusiform face area, extrastriate body area, and fusiform body area) continuously sends off ongoing results of processing into multimodal integrative regions, such as the STS (Campanella and Belin, 2007; Peelen and Downing, 2007; Freeman et al., 2010). There, ongoing visual-processing results of the face begin integrating with ongoing auditory-processing results of the voice, which are emanating from the temporal voice area (Lattner et al., 2005; Campanella and Belin, 2007). While the available bottom-up information (facial, vocal, and bodily cues) begins integrating in multimodal regions such as the STS, the intermediary results of this integration are sent off to higher-order regions, such as the prefrontal cortex (Kim and Shadlen, 1999), in addition to regions involved in decision-making and response selection, such as the basal ganglia (Bogacz and Gurney, 2007). In doing so, bottom-up processing provides tentative support for perceptual alternatives (e.g., some cues provide 75% support for the male category and other cues provide 25% support for the female category; Freeman et al., 2008; Freeman and Ambady, 2011b). The basal ganglia and higher-order regions such as the prefrontal cortex force these partially-active representations (e.g., 75% male and 25% female) to compete, and the ongoing results of this competition are fed back to lower-level cortices where visual and auditory specification is more precise and results can be verified (Treisman, 1996; Bouvier and Treisman, 2010). Before these processing results are fed back, however, they may be slightly adjusted by higher-order regions' top-down biases, e.g., activated stereotypes and motivational states. Lower-level regions then update higher-order regions by sending back revised information (e.g., 85% male and 15% female). Across cycles of this ongoing interaction between the processing of bottom-up sensory cues (instantiated in lower-level regions) and top-down social factors (instantiated in higher-order regions), the entire system comes to settle into a steady state (e.g., ~100% male), presumably reflecting an ultimate, stable perception of another person. This general kind of processing has been captured in a computational model, described below.

### Dynamic interactive model

**Figure 1:**
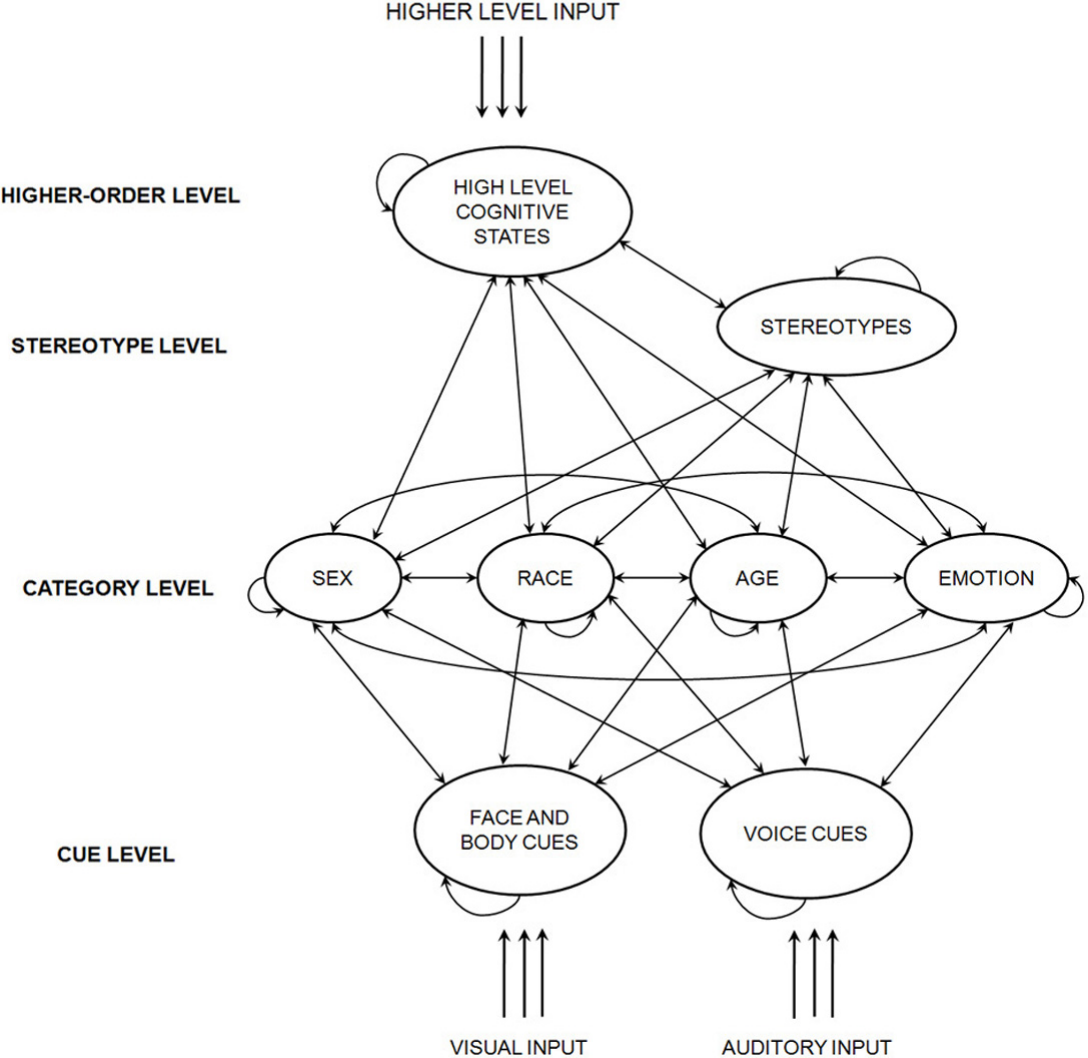
**A general diagram of the dynamic interactive model.** Adapted from Freeman and Ambady (2011a).

A general diagram of the dynamic interactive model appears in Figure 1. It is a recurrent connectionist network with stochastic interactive activation (McClelland, 1991). The figure depicts a number of pools; in specific instantiations of the model, each pool will contain a variety of nodes (e.g., Male, Black, Aggressive, and Female Cues). Specific details on the model's structure may be found in Freeman and Ambady (2011a). The model provides an approximation of the kind of processing that might take place in a human brain (Rumelhart et al., 1986; Smolensky, 1989; Rogers and McClelland, 2004; Spivey, 2007), such as that described above, specifically in the context of perceiving other people.

Initially, the network is stimulated simultaneously by both bottom-up and top-down inputs (see Figure 1). This may include inputs such as visual input of another's face, auditory input of another's voice, or higher-level input from systems responsible for top-down attention, motivations, or prejudice, for example. Each model instantiation contains a variety of nodes that are organized into, at most, four interactive levels of processing (one level representing each of the following: cues, categories, stereotypes, and high-level cognitive states). Every node has a transient level of activation at each moment in time. This activation corresponds with the strength of a tentative hypothesis that the node is represented in the input. Once the network is initially stimulated, activation flows among all nodes simultaneously as a function of their connection weights. Activation is also altered by a small amount of random noise, making the system's states inherently probabilistic. Because many connections between nodes are bi-directional, this flow results in a continual back-and-forth of activation between many nodes in the system. As such, nodes in the system continually re-adjust each other's activation and mutually constrain one another to find an overall pattern of activation that best fits the inputs. Gradually, the flows of activation lead the network to converge on a stable, steady state, where the activation of each node reaches an asymptote. This final steady state, it is argued, corresponds to an ultimate perception of another person. Through this ongoing mutual constraint-satisfaction process, multiple sources of information—both bottom-up cues and top-down factors—are interacting over time toward integrating into a stable perception.^2^

As such, this model captures the intimate interaction between bottom-up and top-down processing theorized here. Thus, together, the approach and model treat perceptions of other people as continuously evolving over fractions of a second and emerging from the interaction between multiple bottom-up sensory cues and top-down social factors. Accordingly, person perception readily makes compromises between the variety of sensory cues inherent to another person and the baggage an individual perceiver brings to the perceptual process. Now, let us consider how this system naturally brings about category interactions such as those described earlier, either through top-down perceiver impacts or bottom-up target impacts.

### Accounting for interactions via top-down impacts

**Figure 2:**
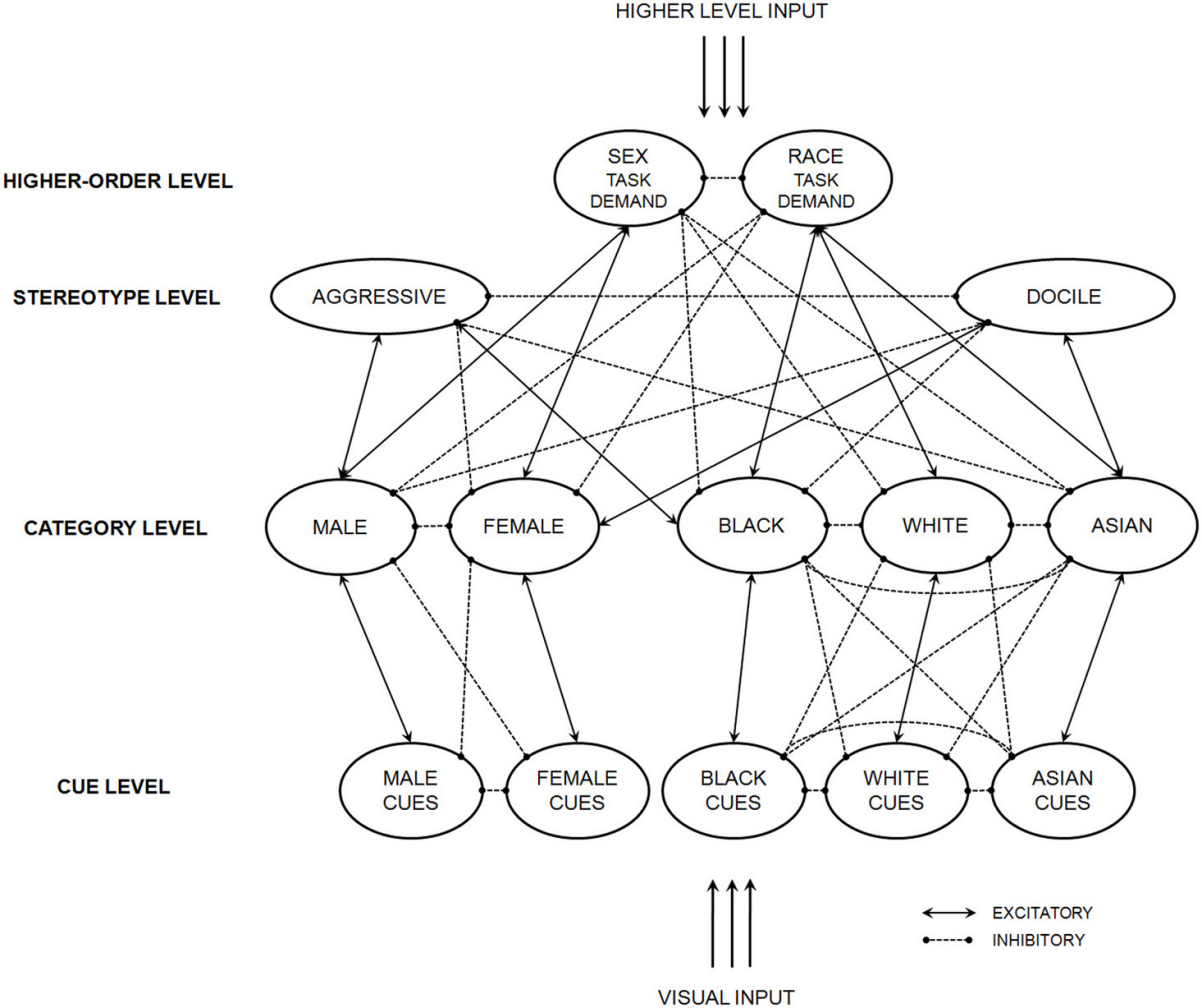
**An instantiation of the dynamic interactive model that gives rise to category interactions driven by top-down stereotypes.** Adapted from Freeman and Ambady (2011a).

A specific instantiation of the general model appears in Figure 2. Solid-line connections are excitatory (positive weight) and dashed-line connections are inhibitory (negative weight). Further details and particular connection weights are provided in Freeman and Ambady (2011a). This instantiation of the model is intended to capture the experience of how a perceiver would go about categorizing either sex or race for a particular task context. When the network is presented with a face, its visual input stimulates nodes in the cue level. Cue nodes excite category nodes consistent with them and inhibit category nodes inconsistent with them. They also receive feedback from category nodes. At the same time that cue nodes receive input from visual processing, higher-level input stimulates higher-order nodes, in this case representing task demands. This higher-level input would originate from a top-down attentional system driven by memory of the task instructions. These higher-order nodes excite category nodes consistent with them, inhibit category nodes inconsistent with them, and are also activated by category nodes as well. Thus, activation of the Race Task Demand node would facilitate activation of race categories (Black, White, Asian) and inhibit activation for sex categories (Male, Female), and vice-versa for the Sex Task Demand node.

One manner by which many categories may interact is through overlapping stereotype contents. For instance, particular social categories in one dimension (e.g., race) may facilitate and inhibit the activation of categories in another dimension (e.g., sex) due to shared activations in the stereotype level. Stereotypes associated with the sex category, male, include aggressive, dominant, athletic, and competitive, and these are also associated with the race category, Black. Similarly, stereotypes of shy, family-oriented, and soft-spoken apply not only to the sex category, female, but also to the race category, Asian (Bem, 1974; Devine and Elliot, 1995; Ho and Jackson, 2001). Thus, there is some overlap in the stereotypes belonging to the Black and male categories and in the stereotypes belonging to the Asian and female categories. Johnson et al. (in press) found that sex categorization was quickened when a computer-generated male face was made to be Black, relative to White or Asian. Conversely, for a female face, sex categorization was quickened when made to be Asian, relative to White or Black. Moreover, when faces were sex-ambiguous they were overwhelmingly categorized as male when Black, but overwhelmingly categorized as female when Asian. Later work found that such influences have downstream implications for interpreting ambiguous identities (e.g., sexual orientation; Johnson and Ghavami, 2011). How could a dynamic interactive model account for such interactions between sex and race, presumably driven by top-down stereotypes?

In the model, category activation along one dimension, e.g., sex, may be constrained by feedback from stereotype activations triggered by the other dimension, e.g., race (see Figure 2). Sex categorization, for example, is constrained by race-triggered stereotype activations. Because the stereotypes of Black and male categories happen to partially overlap, Black men would be categorized more efficiently relative to White and Asian men. As shown in Figure 2, Aggressive happens to be positively linked and Docile happens to be negatively linked with both Black and Male categories. This overlap would lead the race-triggered excitation of Aggressive and race-triggered inhibition of Docile to feed back excitation to the Male category and inhibition to the Female category. This would facilitate a male categorization or, in cases of sex-ambiguous targets, bias categorizations toward male (rather than female). A similar effect would occur with the Asian and Female categories, where race-triggered excitation of Docile and race-triggered inhibition of Aggressive would come to facilitate a female categorization or bias categorizations toward female. Thus, a dynamic interactive model predicts that incidental overlap in stereotype contents could powerfully shape the perception of another category dimension.

When actual simulations were run with the network appearing in Figure 2, it was found that race category was readily used to disambiguate sex categorization. When a sex-ambiguous face was Black, the network was biased toward male categorization, with a 26% likelihood to categorize it as female. When White, random noise seemed to be driving sex categorization one way or the other, with a 52% likelihood (random chance: 50%) of female categorization. When Asian, however, the network was biased toward female categorization, with a 75% likelihood of female categorization (Freeman and Ambady, 2011a). Thus, a dynamic interactive model predicts that perceivers would be biased to perceive sex-ambiguous Black faces as men and, conversely, to perceive sex-ambiguous Asian faces as women. This is because the presumably task-irrelevant race category placed excitatory and inhibitory pressures on stereotype nodes which were incidentally shared with sex categories. Indeed, Johnson et al. (in press) obtained this precise pattern of results with human perceivers.

### Accounting for interactions via bottom-up impacts

As discussed earlier, different categories may be thrown into interaction because the perceptual cues supporting those categories partly overlap and are therefore directly confounded. For instance, sex categorization is facilitated for faces of happy women and angry men, relative to happy men and angry women. Further studies solidified the evidence that this interaction between sex and emotion is due to direct, physical overlap in cues rather than merely top-down stereotypes (see also Becker et al., 2007; Hess et al., 2009; Oosterhof and Todorov, 2009). Thus, these studies suggest the features that make a face angrier are also partly those that make a face more masculine. Similarly, the features that make a face happier are also partly those that make a face more feminine. For instance, anger displays involve the center of the brow drawn down-ward, a compression of the mouth, and flared nostrils. However, men also have larger brows which may cause them to appear drawn down-ward. They also have a more defined jaw and thinner lips, which may make the mouth to appear more compressed, and they have larger noses, which may lead to the appearance of flared nostrils. A similar overlap exists for happy displays and the female face (Becker et al., 2007). For instance, women have rounder faces than men, and the appearance of roundness increases when displaying happiness (i.e., a smile draws out the width of the face). Previous studies suggest that it is this direct, physical overlap in the cues signaling maleness and anger and in the cues signaling femaleness and happiness that leads to more efficient perceptions of angry men and happy women (relative to happy men and angry women).

**Figure 3:**
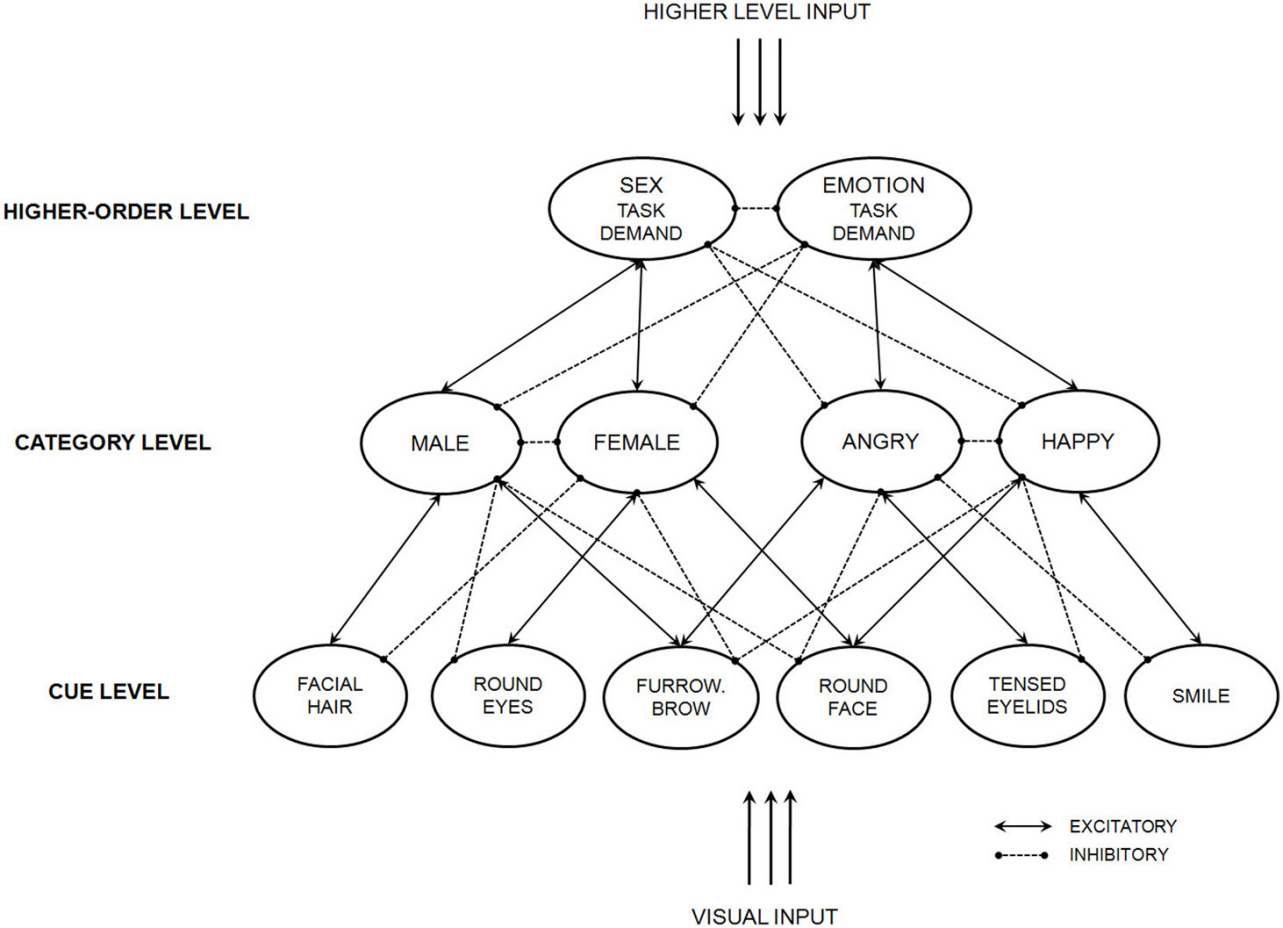
**An instantiation of the dynamic interactive model that gives rise to category interactions driven by bottom-up perceptual cues.** Adapted from Freeman and Ambady (2011a).

A second instantiation of the general model appears in Figure 3 (particular connection weights found in Freeman and Ambady, 2011a). Differing from the previous instantiation, here nodes in the cue level represent a single perceptual cue (e.g., defined jaw and smile). Note that one cue node, Facial Hair, has an excitatory connection with Male and inhibitory connection with Female, whereas another cue node, Round Eyes, has an excitatory connection with Female and inhibitory connection with Male. Similarly, one cue node, Tensed Eyelids, has an excitatory connection with Anger and inhibitory connection with Happy, and vice-versa for the cue node, Smile. These four cue nodes represent the perceptual cues that independently relate to sex categories and independently relate to emotion categories. However, also note that one cue, Furrowed Brow, has an excitatory connection both with Anger and with Male (since a furrowed brow conveys both categories, described earlier). Similarly, another cue, Round Face, has an excitatory connection both with Happy and with Female (since a rounder face conveys both categories, described earlier). Thus, these two cue nodes represent the bottom-up overlap in the perceptual cues conveying sex and emotion. Specific cues used in this simulation were chosen arbitrarily; they are merely intended to simulate the set of non-overlapping and overlapping perceptual cues that convey sex and emotion.

When actual simulations were run with the network, the overlapping perceptual cues created bottom-up pressure that give rise to interactions between sex and emotion. When a male face was angry, the Male category's activation grew more quickly and stabilized on a stronger state, relative to when a male face was happy. Conversely, however, when a female face was angry, the Female category's activation grew more slowly and stabilized on a weaker state, relative to when a female face was happy. This led sex categorization of angry men and happy women to be completed more quickly (Freeman and Ambady, 2011a). This pattern of results converges with the experimental data of previous studies (Becker et al., 2007). Thus, the categorization of one dimension (e.g., sex) may be shaped by direct bottom-up overlap with the perceptual features supporting another dimension (e.g., emotion). This highlights how the model naturally accounts for such category interactions driven by bottom-up perceptual overlaps.

## Conclusion

One of the most fascinating aspects of person perception, which distinguishes it from most kinds of object perception, is that a single social percept can simultaneously convey an enormous amount of information. From another's face, multiple possible construals are available in parallel, including sex, race, age, emotion, sexual orientation, social status, intentions, and personality characteristics, among others. Here we have reviewed two manners by which many of these construals may interact with one another. One manner is through top-down perceiver impacts, where existing knowledge structures, the stereotypes a perceiver brings to the table, motivations, and other social factors throw different dimensions into interaction. Another manner is through bottom-up target impacts, where the perceptual cues supporting different dimensions are inextricably linked, leading those dimensions to interact. Further, these interactions in person perception may often occur in functionally adaptive ways. We then discussed a recent computational model of person perception that we argued is able to account for many of these sorts of interactions, both those driven by top-down and bottom-up forces. In short, person perception is combinatorial, and treating our targets of perception as having multiple intersecting identities is critical for an accurate understanding of how we perceive other people. Research investigating the underlying mechanisms of person perception is growing rapidly. To take up this new level of analysis in understanding person perception successfully, collaboration between scientists in traditionally divided domains is needed, such as the social-visual interface (Adams et al., 2011). Here, we have argued that there is a coextension among sensory and social processes typically investigated independently. To map out how low-level visual information (traditionally home to the vision sciences) may meaningfully interact with and be shaped by high-level social factors (traditionally home to social psychology), and how this is instantiated through all the cognitive and neural processing lying in between them, interdisciplinary collaboration will be important. The emerging study of social vision offers an exciting and multilevel approach that may help bring about a more unified understanding of person perception. At the same time, it provides a unique bridge between far-reaching areas of the field, from researchers in social psychology to the cognitive, neural, and vision sciences.

### Conflict of interest statement

The authors declare that the research was conducted in the absence of any commercial or financial relationships that could be construed as a potential conflict of interest.

## Footnotes

^1^It is important to note that such findings could also potentially be accounted for by a response bias. In such a case, the top-down effect may be the result of a higher-order decision-level response criterion that is altered by stereotypic expectations or a perceiver' motivational state, rather than a genuine change in perception occurring.

^2^Given that the connectionist model employs localist nodes that are relatively abstract and that model parameters are hand-coded, the model should be seen as an existence proof for the kind of processing proposed. Future research will need to expand and refine the model to more rigorously investigate its plausibility.
